# Effects of the Chemical Treatment on the Physical-Chemical and Electrochemical Properties of the Commercial Nafion™ NR212 Membrane

**DOI:** 10.3390/ma13225254

**Published:** 2020-11-20

**Authors:** Enza Passalacqua, Rolando Pedicini, Alessandra Carbone, Irene Gatto, Fabio Matera, Assunta Patti, Ada Saccà

**Affiliations:** National Research Council, Institute for Advanced Energy Technologies “Nicola Giordano” (CNR-ITAE), via Santa Lucia Sopra Contesse, 5, 98126 Messina (ME), Italy; rolando.pedicini@itae.cnr.it (R.P.); alessandra.carbone@itae.cnr.it (A.C.); irene.gatto@itae.cnr.it (I.G.); fabio.matera@cnr.it (F.M.); assunta.patti@itae.cnr.it (A.P.); ada.sacca@itae.cnr.it (A.S.)

**Keywords:** chemical treatments, membrane degradation, nafion™ NR212, polymer electrolyte fuel cells, polymer exchange membranes, water uptake

## Abstract

Polymer Electrolyte Fuel Cells (PEFCs) are one of the most promising power generation systems. The main component of a PEFC is the proton exchange membrane (PEM), object of intense research to improve the efficiency of the cell. The most commonly and commercially successful used PEMs are Nafion™ perfluorosulfonic acid (PFSA) membranes, taken as a reference for the development of innovative and alternative membranes. Usually, these membranes undergo different pre-treatments to enhance their characteristics. With the aim of understanding the utility and the effects of such pre-treatments, in this study, a commercial Nafion™ NR212 membrane was subjected to two different chemical pre-treatments, before usage. HNO_3_ or H_2_O_2_ were selected as chemical agents because the most widely used ones in the procedure protocols in order to prepare the membrane in a well-defined reference state. The pre-treated membranes properties were compared to an untreated membrane, used *as-received*. The investigation has showed that the pre-treatments enhance the hydrophilicity and increase the water molecules coordinated to the sulphonic groups in the membrane structure, on the other hand the swelling of the membranes also increases. As a consequence, the untreated membrane shows a better mechanical resistance, a good electrochemical performance and durability in fuel cell operations, orienting toward the use of the NR212 membrane without any chemical pre-treatment.

## 1. Introduction

Polymer Electrolyte Fuel Cells (PEFCs) are emerging as attractive energy conversion systems suitable for use in many industrial applications, starting from a few milliwatts for portables to several kilowatts for stationary and automotive applications [1,2,3,4,5].

A PEFC consists of a solid polymeric electrolyte (Proton Exchange Membrane, PEM) between two porous electrodes: an anode and a cathode, where the electrochemical reactions occur. The hydrogen gas and oxygen gas are continuously fed to the anode and cathode, respectively. At the anode, the hydrogen fuel reacts with a catalyst, generating positively charged protons (H^+^) and negatively charged electrons (e^−^). The electrolyte membrane allows the positive ions to flow from the anode to the cathode side while the electrons pass through an external electrical circuit to the cathode. Simultaneously, on the cathode side, there is the water formation as a result of the reaction between the oxygen and electrons from the electrode and protons from the electrolyte [6]. However, the main components of a PEFC (PEM, gas diffusion electrodes, bipolar plates, etc.) are still the object of an intense technological research, especially the PEM, which is the core of the fuel cell [7,8,9,10].

PEM has the function of: (I) separator in order to prevent the mixing of anodic and cathodic reactants; (II) conductor for the protons from the anode to cathode; (III) electrical insulator to drive the electrons through the external circuit to the cathode. It should meet the following requirements: (I) high ionic conductivity; (II) low fuel permeability; (III) good thermal and hydrolytic stability; (IV) good electrochemical stability in aggressive environments; (V) morphological and dimensional stability; (VI) considerable mechanical properties in both dry and hydrated states [6].

The most commonly used PEMs in fuel cells are the well-known perfluorosulfonic acid (PFSA) membranes. PFSA based polymers are composed of an high ly hydrophobic perfluorinated polytetrafluoroethylene (PTFE) like backbone and highly hydrophilic sulfonic acid functional groups in side chains attached by an ether group. In presence of water, the sulfonic acid functional groups aggregate to form a hydrophilic domain. When this is hydrated, protonic charge layers due to the dissociation of the acidic functional groups and proton conductance assisted by water dynamics occur. While the well-connected hydrophilic domain is responsible of the transport of protons and water, the hydrophobic domain provides to the polymer the morphological stability and prevents the polymer from dissolving in water. Among PFSA membranes, the most studied and commercially used are Nafion™ based membranes. It is an ionomer developed by DuPont de Nemours & Co since 1960s and now manufactured by The Chemours Company [11], a DuPont spin-off. The most used Nafion™ membranes are long side chain (LSC) membranes (Equivalent Weight of 1100), both extruded membranes (N117, N115, N1110) and membranes from cast solution (NR211 and NR212). They vary in terms of thickness, from 89 to 254 µm for the NE (Nafion Extruded) and from 25 to 50 µm for NR (Nafion Recast), respectively [12,13].

Before of the characterization on commercial Nafion™ membranes, in literature it is often suggested to follow chemical or thermal pre-treatments with the aim of casting the membranes in a well-defined state. Different pre-treatments are reported in literature for preparation of acid-form (-RSO3H) membranes. Such membranes exist in several states—*as-received* (un-treated), typical of the ending product; *expanded-state*, after consequential treatments in boiled acid and water; *shrunk-state*, obtained by desiccation at temperatures close to Glass Transition Temperature (Tg > 100 °C) and *normal or reference-state*, achieved by conditioning and desiccation treatments at low temperature.

Among all these states, the chemical pre-treatments, aimed to oxidize the impurities coming from the fabrication process and/or to enhance the hydrophilicity of the membranes, could produce some discrepancies in Nafion™ membranes properties [14].

In this paper, two different chemical treatments (by HNO_3_ or H_2_O_2_) were studied with the aim of reporting to a reference state a commercial Nafion™ NR212 membrane before of its usage. The results were compared to an untreated membrane. The investigation has regarded the influence of pre-treatments on water absorption, Ion Exchange Capacity (IEC), mechanical properties and so forth. Moreover, the electrochemical behavior in fuel cells has been evaluated in term of both performance and durability through Accelerated Stress Tests (ASTs), to understand whether the pre-treatments have an effect on the membrane durability. The aim of the study is to verify if the acidic and oxidative pre-treatments can give a prompt enhancement of membranes properties, such as wettability and acidic properties but if at the same time can weaken the structure affecting their duration during fuel cell operations.

## 2. Materials and Methods 

### 2.1. Materials and Membranes Treatments

Nafion™ (a brand of the Chemours Company formerly DuPont) NR212 commercial membrane, purchased from Ion Power^®^ (New Castle, DE, USA), was used in this study. The characteristics of membrane, as declared from the supplier, are reported in Table 1:

Such a membrane was studied *as-received* and treated by two different chemical treatments, in order to evaluate the effects in terms of chemical-physical and electrochemical properties. HNO_3_ or H_2_O_2_ were used as chemical agents.

When HNO_3_ is used as an oxidant agent, the *as-received* membrane was immersed in an aqueous solution (about 7M) of HNO_3_ (HNO_3_:H_2_O = 1:1) at 80 °C for 30 min, then it was boiled in distilled water for 15 min. Successively, the membrane was immersed in H_2_SO_4_ 1M at 80 °C for 30 min and three final washings in boiling water were performed for 15 min each.

When H_2_O_2_ is used, the original membrane was immersed in an aqueous H_2_O_2_ (5 vol.%) solution at 80 °C for 30 min., then rinsed different times in distilled water and successively it was treated in a boiling solution of H_2_SO_4_ 1M for 30 min. Also, in this case, the acid traces were completely removed by three successive washings in boiled water of 15 min each.

Considering *as-received* and treated membranes, they will be successively identified in the paper as N-AR, N-HNO3 and N-H2O2.

### 2.2. SEM Analysis

The morphology on untreated and treated commercial membranes was observed through cross-section SEM measurements using a Field Emission Scanning Electron Microscope (FE-SEM model XL30 S FEG, Philips, Amsterdam, NL, USA) at different magnifications using a low electron beam (5 kV). Membranes for cross-section measurement were prepared using freeze-dry cut procedure by dipping in liquid Nitrogen (N_2_) and breaking the samples to have a perfect fracture. A gold coating was used to avoid sample charging and to permit electronic conduction.

### 2.3. Chemical-Physical Characterizations

Water uptake (*Wup*) was determined according to the following Equation [15]:

*Wup* (%) = [(m_wet_ − m_dry_)/m_dry_] × 100 (1)

In Equation (1), m_wet_ and m_dry_ are the wet and dry weights of the membrane. The m_dry_ was measured after desiccation of the samples in an oven under vacuum at 80 °C for 2 h, while m_wet_ was measured in three different conditions, soaking the membrane in: (1) distilled water at room temperature (rT) for 24 h, (2) at 80 °C for 2 h, (3) at 95 °C for 2 h. In these same conditions, dimensional variations were calculated in terms of thickness and area percentage variation, using similar formulas to the water uptake:

A (%) = [(A_wet_ − A_dry_)/A_dry_] × 100 (2)

T (%) = [(T_wet_ − T_dry_)/T_dry_] × 100 (3)

In Equations (2) and (3), A_wet_, A_dry_ and T_wet_, T_dry_ represent the area and thickness dimensional parameters in wet and dry conditions.

Such parameters were used for the volume variation determination and, hence, to evaluate the *Swelling* (S) of the membrane, calculated by the ratio of wet volume and dry volume (*V*_wet_/*V*_dry_) for each sample, at the corresponding above-mentioned conditions.

The experimental Ion Exchange Capacity (IEC) [15], expressed as meq_-SO3H_·g^−1^, was determined by an acid-base titration with an automatic titrator Model 751 GPD Titrino (Metrohm AG, Herisau, Switzerland) on the previously dried samples at 80 °C for 2 h under vacuum. The membranes were soaked in 1 M NaCl solution to exchange the H^+^ of the -SO_3_H groups with Na^+^ and the solution containing the exchanged H^+^ was titred with a 0.01 M NaOH solution. The IEC values were calculated using the following Equation:

IEC = (V_NaOH_ × M)/m_dry_ (4)

V_NaOH_ (mL) is the added titrant volume at the equivalent point; M is the molar concentration of the titrant.

The λ value (expressed as moles_H2O_ and moles_-SO3H_ ratio) was calculated through the *Wup* and IEC values ratio, both expressed in moles, as described in Equation (5):

λ = n_H2O_/n_-SO3H_ = [(*Wup*/100)/MW_H2O_] × 1000/IEC (5)

where MW_H2O_ is the water molecular weight equal to 18.

For chemical-physical characterizations, the measurements were repeated three times in order to have a statistical validity.

### 2.4. Mechanical Characterizations

The mechanical properties of the membranes were evaluated through a Dynamic Mechanical Analyzer Model DMA1 (Mettler Toledo, Columbus, OH, USA) using the tension clamp configuration for like-film/fiber samples. A preload force of 0.1 N was preventively applied to softly trend the samples before of the measurement. By a Software (Star Software V12, Mettler Toledo, Columbus, OH, USA), the spectra were measured by subjecting a rectangular film sample of 10 mm (length) × 5 mm (width) to an oscillatory sinusoidal tensile deformation at 1 Hz imposing the displacement control with an amplitude of 20 µm [16]. Measurements were carried out in the temperature range between 30 to 150 °C at a rate of 5 °C·min^−1^. The mechanical response of materials was analyzed in terms of the elastic storage (E’) and viscous loss modulus (E”). Tan δ (E” and E’ ratio) was analyzed to measure the Glass Transition Temperature (Tg) of the membranes investigated.

### 2.5. Electrochemical Characterisations

The proton conductivity (PC) measurement on membranes was carried out by using a commercial conductivity PTFE cell (Bekktech, LLC acquired by Scribner Associates Inc. in 2011, Southern Pines, NC, USA)in the longitudinal direction by the four-probes method at T_cell_ = 80 °C and fully humidification (100% RH), P = 1 atm. with a hydrogen flux of 1000 sccm, in Direct Current (DC). The cell is connected to a test station and a potentiostat-galvanostat mod.551 (AMEL S.r.L., Milano, Italy) [16]. A membrane sample of about 2.5 cm × 0.52 cm was cut by a sample punch. Its area was measured by a width measurement tool (by BekkTech), while the thickness was measured by a electronic gauge (Mitutoyo Italiana S.r.L, Milano, Italy). The membrane was assembled in the cell and placed in contact with the two fixed platinum electrodes. By an indirect imposition of the current, a voltage drop between the two fixed electrodes was measured. Electrical resistance averaged values in the different current ranges (at least three R values for each current range) were obtained by extrapolating the data from the plot of current as a potential function. At the end, the PC (σ, S·cm^−1^) was calculated using the following formula:

σ = L/(R × W × T)(6)

where L = 0.425 cm, fixed distance between the two Pt electrodes; R = resistance in Ω; W = sample width in cm; T = sample thickness in cm. The measurement was carried out in continuous for about 80 h. by checking the conductivity value every 8 or 12 h.

Gas Diffusion Electrodes (GDEs) were prepared by spraying the Catalyst Layer (CL) ink on a Sigracet SGL 24 BC carbon paper, following a standardized procedure [17]. The CL ink was composed of 33 wt.% of dry Nafion^®^ (5 wt.% hydroalcoholic solution by Aldrich), 50 wt.% Platinum on Carbon (Pt/C, by Alfa Aesar) as the electro-catalyst and an aqueous solution of NH_4_CO_3_ to enhance the CL porosity. The same amount of Pt (0.5 mg_Pt_·cm^−2^) was applied on both anode and cathode. GDEs were hot pressed (P = 20 Kg·cm^−2^) on the membranes at 125 °C for 5min to form the corresponding Membrane Electrodes Assemblies (MEAs), using PTFE gaskets of 0.2 mm for the sealing of MEAs.

The MEAs were characterized in a 25 cm^2^ single cell fed by H_2_/air, connected to a commercial test station (Fuel Cell Technologies. Inc., Albuquerque, NM, USA) and equipped of a suitable software (version FCT_Programs2011-10-20) for the data acquisition.

The polarization curves were carried out in conventional conditions (Tcell = 80 °C and RH = 100%) at 1.5 abs. bar. A constant stoichiometry of 1.5 times and 2 times for the fuel and the oxidant was used, respectively. Before of each test, MEAs were conditioned at Tcell = 80 °C, 100%RH and 3 abs. bar. maintaining the cell at a constant potential (0.6 V) for 7 h.

In-situ electrochemical degradation of the MEAs were carried out by Accelerated Stress Tests (named ASTs), in the same operative conditions, by cycling the current density between 0.1 and 0.4 A·cm^−2^ (30 min for each current density, therefore each cycle lasted 60 min) simulating the swelling-deswelling process. ASTs were carried out for about of 250 h. After each series of approximately 24 cycles, a check of Open Circuit Voltage (OCV) value was carried out.

The cell was connected to the fuel cell test station equipped with a HP6051A electronic load and an AUTOLAB Metrohm Potentiostat/Galvanostat with a Frequency Response Analyser (FRA) module and a 20A current booster. The Ionic Resistance (Rs) was calculated by impedance spectroscopy (EIS) curves recorded under the same operative conditions of I–V curves. All the impedance measurements were performed in the potentiostatic mode at 700mV, in the frequency range from 0.1 Hz to 100 kHz with an amplitude of the signal perturbation of 10 mV.

H_2_ crossover measurements on MEAs were performed by Linear Sweep Voltammetry (LSV) method, using potentiostat-galvanostat device (Autolab) and feeding the cell by fully humidified (100%RH) pure H_2_ and N_2_ at the anode and cathode side at 1.5 abs. bar, respectively. The potential was scanned from 0.0 V up to 0.8 V with a scan rate of 4mV·s^−1^. The current value recorded at 400 mV was used for the H_2_ crossover calculation.

I–V curves acquisition, H_2_ crossover determination and EIS were recorded at the Beginning of Test (BoT), at about 150–170 h and at End of Test (EoT).

## 3. Results and Discussion

Nafion™ NR212 is a membrane based on chemically stabilized perfluorosulfonic acid (PFSA)/polytetrafluoroethylene (PTFE) copolymer in the acid (H^+^) form. The perfluorinated backbone provides chemical and mechanical stability, the ether groups provide flexibility, while the sulfonic acid groups yield high ionic conductivity. It is prepared through a casting procedure with a thickness of 50.8 µm [13]. Even if an extrusion method is not used for the membrane fabrication, some treatments are often carried on the as received membranes to oxidize the impurities stored during the fabrication process or to enhance the membrane hydration and protonation [18].

**Figure 1:**
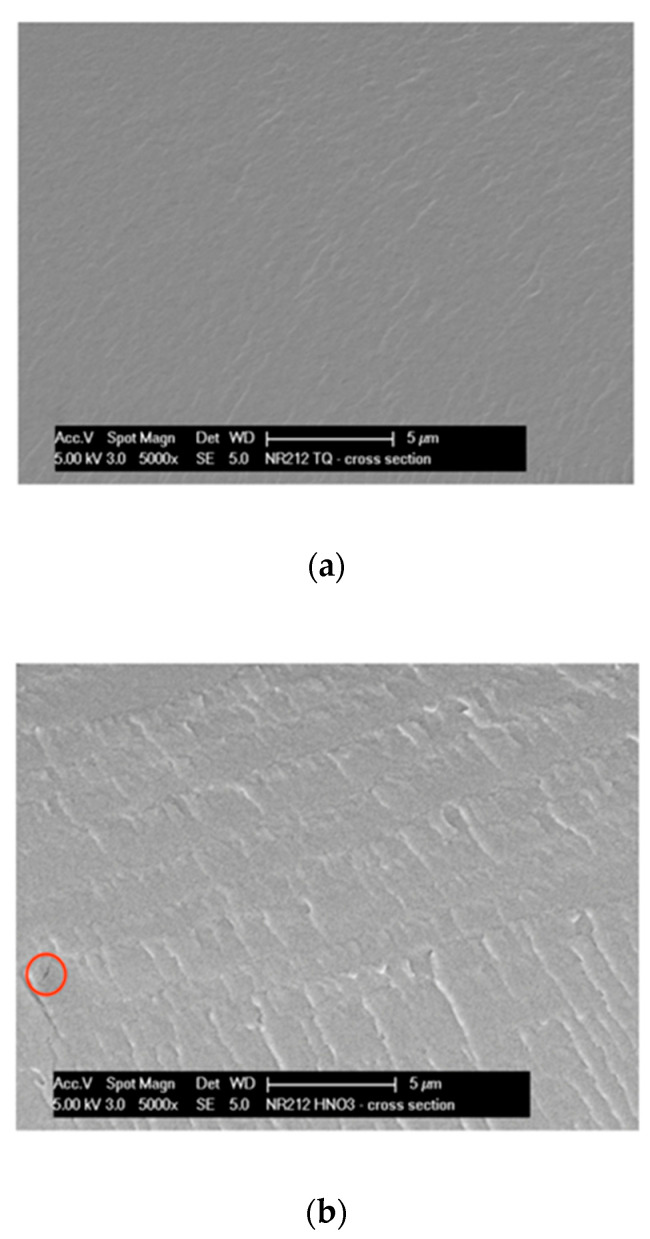

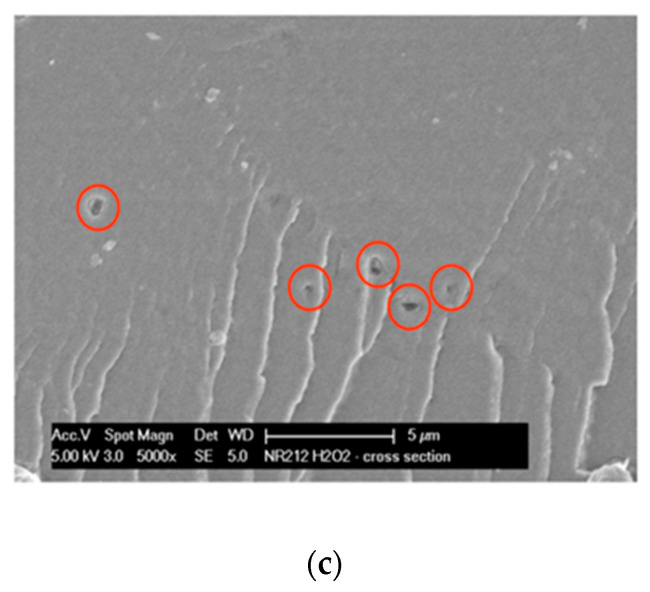
Comparison of membranes cross-section scanning electron microscopic (SEM) images: (**a**) N-AR, (**b**) N-HNO3, (**c**) N-H2O2.

Although in literature [19], no particularly relevant morphological difference among differently treated N117 membranes was found from scanning electron microscopic (SEM) images, the NR212 membranes, studied in the present work, were analyzed through SEM analysis. Figure 1a–c reports the comparison of the cross-section SEM images obtained on untreated and treated membranes at 5000 × magnification.

The as-received sample N-AR (a) maintains a good morphology under the electron beam; differently, at the same magnification, both treated membranes supply evidence of an important damage under the beam. In particular, the N-HNO3 membrane (b) shows an important dark area with a corresponding tear, indicated from a red circle; while, the N-H2O2 membrane (c) shows some holes, indicated from red circles. This demonstrates as the as-received membrane resists better to electron beam than the treated membranes, in general confirming a good mechanical resistance.

The declared available acid capacity of the membrane is 0.92 meq·g^−1^, while the total acid capacity is 0.95–1.01 meq·g^−1^. This means that a further acid treatment could increase the acid capacity from about 3% to 6%. In Table 2, we report the experimentally measured IEC values for the *as-received* and pre-treated membranes.

The experimental values are higher than the available acid capacity declared from Ion Power (Table 1) and they approach the total acid capacity for all the membranes. HNO_3_ is a weaker acid respect to the sulfonic acid group of Nafion^™^ and the higher pKa value makes the protonation incomplete. Moreover, it does not allow to exploit the total acid capacity of the polymer, hence leading to a lower IEC value if compared to the un-treated membrane [20]. When NR212 is immersed in a 5 vol.% H_2_O_2_ solution, even if for a very short time, a probable decomposition of the C–F bonds and the sulfonic acid groups of H^+^ Nafion occurs with a decomposition ratio of the sulfonic acid groups higher than that of the C–F bond [21,22]. This fact could be responsible of the slight IEC decrease in N-H2O2 with respect to N-AR.

**Figure 2:**
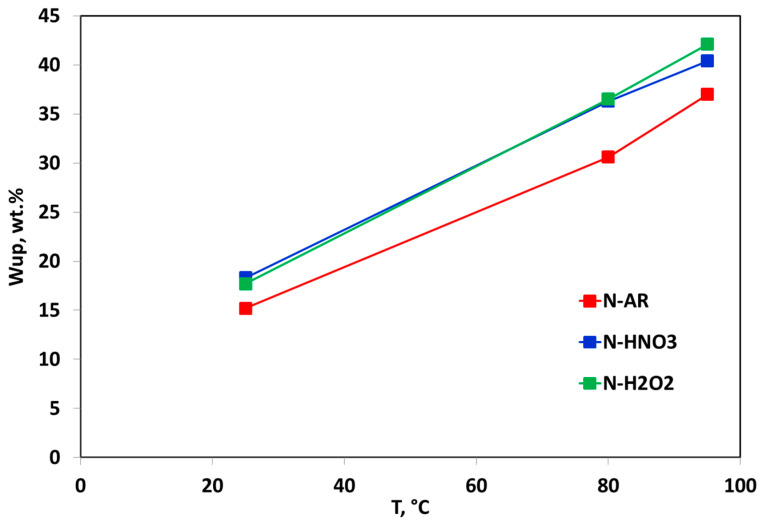
Comparison between *Wup* measured in liquid water at rT, 80 °C, 95 °C for N-AR (red squares), N-HNO3 (blue squares) and N-H2O2 (green squares) membranes.

**Figure 3:**
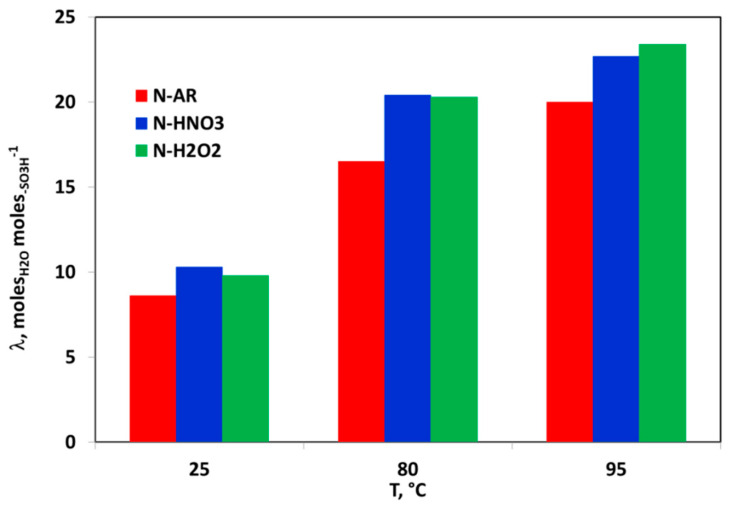
Number of water moles for sulfonic group (λ) as a function of temperature for N-AR (red bar), N-HNO3 (blue bar) and N-H2O2 (green bar).

Different models have been proposed to explain the structure of the Nafion™ membranes [23,24,25] but, although these models differ in the geometry and spatial distribution of the ionic clusters, they agree that exists a phase separation between the hydrophobic backbone and hydrophilic sulfonic-acid side chains in which the water clusters are located. The size and shape of the hydrophilic clusters strongly depend on the water content in the material, therefore all of the transport properties of polymeric membranes depend on their hydration. In particular, the protonic conductivity is dramatically affected by the amount of water that is absorbed by the membrane, therefore it is very important to measure or calculate the degree of hydration or water content. The *Wup* and the water amount associated to the sulfonic groups, expressed as λ, are the main parameters related to water content. In Figure 2 and Figure 3, *Wup* and λ as a function of temperature are reported, respectively.

Both values increase with the temperature increase. *Wup* (Figure 2) for the pre-treated membranes is very high if compared to N-AR membrane and consequently the water molecules associated to sulfonic groups (Figure 3) also increase. The pre-treatments carried out at 80 °C by both HNO_3_ and H_2_O_2_ increase the hydrophilic properties of the membrane and lead to gain more molecules of water with respect to the untreated membrane [20,26]. A λ value ≤ 3 indicates that the water molecules are in the primary hydration sphere. The first absorbed water molecules cause the dissociation of the sulfonic group, resulting in the formation of hydronium ions. All water molecules absorbed by the membrane at this low water content are associated with the sulfonic groups. As the amount of water absorbed is insufficient for the formation of a continuous water phase, so the conductivity will be extremely low. In the range of water loosely bound (13 < λ < 16), the counterion clusters continue to grow, while the excess of charge proton is mobile over the entire cluster. For higher values of λ (water as second phase), the counterion clusters coalesce to form larger clusters and a continuous phase is formed with properties approaching those of bulk water [27]. For the *as-received* and pre-treated membranes, at rT the absorbed water is loosely bound water, while from 80 °C upwards, the absorbed water is a second phase water, favoring the proton conducting.

**Figure 4:**
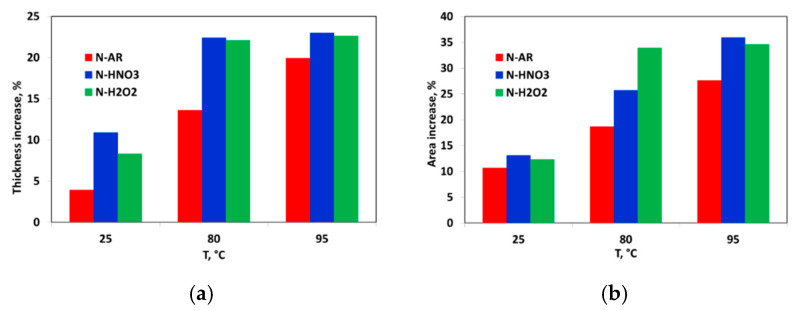
(**a**) Percentage variation of thickness; (**b**) Percentage variation of area from dry to wet state for N-AR (red bar), N-HNO3 (blue bar) and N-H2O2 (green bar) as a function of temperature.

In order to verify the effect of water absorption on the dimensional variations, the expansion of the membranes in liquid water has been analyzed in terms of the percentage increase of thickness and area. The results are reported in Figure 4a,b. Since the plotted values are an average value on three different samples, the Standard Deviation has been calculated and it has found to be equal to 1–1.5 for %Thickness (Figure 4a) and 2 for %Area (Figure 4b).

The temperature increase produces an increase of both parameters. The dimensional variations in the in-plane direction of the membranes can lead to a delamination between the membrane and the electrodes during fuel cell operation, while an increase in the thickness direction can result in an increased pressure between the MEA and the bipolar plates that can be compensated by compression of the MEA. For this reason, the thickness variation has a smaller impact on the cell failure [28]. Coherently to *Wup* and λ results, also in this case, a similar trend was evidenced. Pre-treated membranes show a major percentage increase of thickness and area than *as-received* membrane due to the higher hydrophilicity.

**Figure 5:**
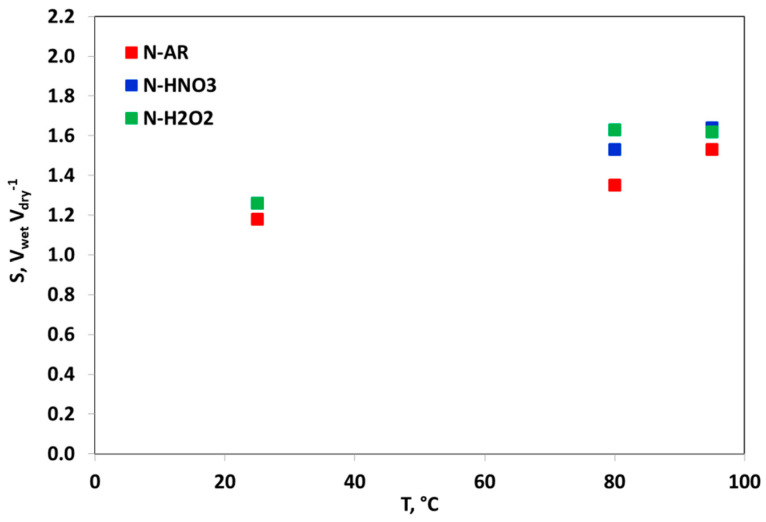
Influence of the liquid water temperature on the swelling of N-AR (red squares), N-HNO3 (blue squares) and N-H2O2 (green squares) membranes.

This trend has an impact on the membranes swelling *S*. The *S* behavior is shown in Figure 5. It is desirable that membranes do not excessively swell in hot liquid water, because the volume swelling in fuel cell operative conditions can cause an excessive mechanical force affecting the durability of the entire MEA. For this reason, a swelling of 2 is considered as a limit to be not overpassed [29]. Usually, Nafion^®^ membranes have a restrained *S* value at T < 100 °C but a pre-treatment by HNO_3_ and H_2_O_2_ increases this value, especially at high temperature.

**Figure 6:**
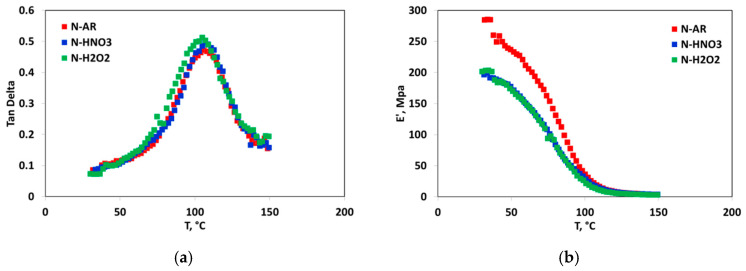
(**a**) tan δ and Storage modulus trend as a function of the temperature of N-AR (red squares), N-HNO3 (blue squares) and N-H2O2 (green squares) membranes.

The mechanical stability of the membranes was investigated by DM analysis. In Figure 6a,b, tan δ behavior as a function of temperature and the storage modulus (E’) behavior into the investigated range (rT–150 °C) are reported, respectively.

N-AR and N-HNO3 membranes have the same T_g_ value (106 °C), while the membrane treated with H_2_O_2_ shows a slighter low T_g_ value (104 °C). Moreover, a higher mechanical resistance for un-treated membrane confirms a lower vulnerability of this membrane respect to the pre-treated ones. Similarly, Tang et al. [30], comparing the Young’s modulus for *as-received* N112 membrane to pre-treated membranes in boiling H_2_O_2_ and H_2_SO_4_, found that the pre-treatments promote the creation and growth of the hydrated ionic clusters within the structure, with a consequent higher water uptake and lower Young’s modulus.

**Figure 7:**
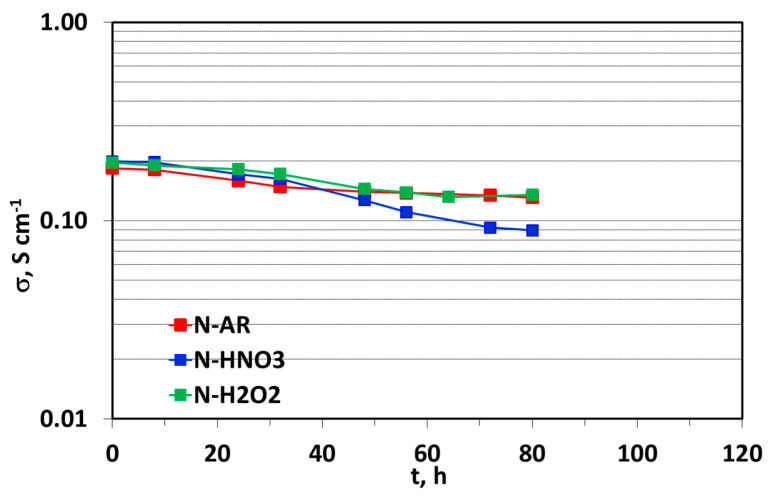
Proton Conductivity at 80 °C and 100% RH as a function of time.

The in-plane PC measurements were performed at 80 °C and 100% RH as a function of the time and the results are shown in Figure 7.

In the first 40 h, the PC for treated membranes is higher than N-AR sample. This behavior was predictable because the chemical treatments enhance the wettability and the acidic properties of the membranes. After this time, a change of trend is detectable with an evident decrease of PC for treated membranes, especially for N-HNO3 and a good stability for un-treated membrane. The PC loss rate calculated within the 80 h was: 0.69, 0.39 and 0.36 S·cm^−1^·h^−1^ for N-HNO3, N-H2O2 and N-AR, respectively.

These results confirm an initial higher PC value for treated membranes with respect to un-treated one but this advantage is reduced with the time. It would be interesting to deepen this behavior, therefore this study will be extended on other casted or extruded PFSA and non PFSA membranes.

**Figure 8:**
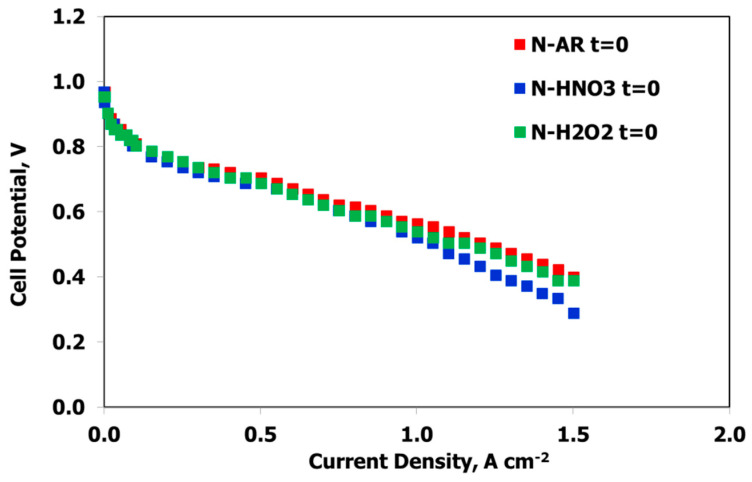
I–V curves at BoT (t = 0) at 80 °C, 100% RH, 1.5 abs bar. for N-AR (red squares), N-HNO3 (blue squares), N-H2O2 (green squares).

MEAs fabricated with the three membranes were electrochemically characterized in a single fuel cell at conventional conditions (80 °C, 100% RH, 1.5 abs bar) with the aim of evaluating the influence of the treatments on the polarization curves and the degradation tests. In Figure 8, the I–V curves at BoT are reported.

**Figure 9:**
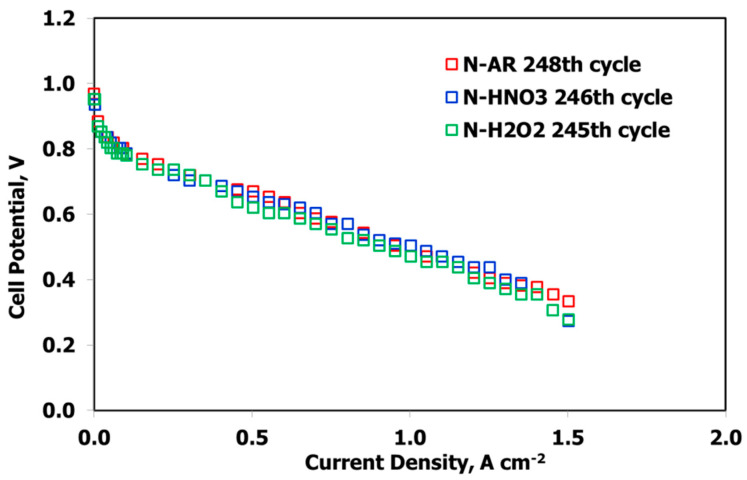
I–V curves at EoT at 80 °C, 100% RH, 1.5 abs bar: 248 cycles for N-AR (red squares), 246 cycles for N-HNO3 (blue squares), 245 cycles for N-H2O2 (green squares).

The curves are sufficiently superimposable in the activation region; a slight improvement in the ohmic region is evident for N-AR and this is due to the higher IEC value with respect to the other two membranes. After about 250 cycles of degradation test (EoT), the curves are almost similar (Figure 9), showing no drastic loss.

In order to better understand the differences in the degradation trend, the electrochemical values at three different current densities are reported in Table 3. Δ is the percentage loss of cell potential between BoT and EoT.

In the activation region, Δ is very low and similar for all of the membranes; this region, in-fact, measures the catalyst effectiveness and, in this work, the same catalyst was used.

In the ohmic region, the cell potential loss includes the electronic and ionic contributions to fuel cell resistance but the ionic contribution, related to the ionic resistance of the membrane, dominates the reaction since the ionic transport is more difficult than the electronic charge transport. In this case, some differences between the differently treated membranes are evidenced. N-H2O2 is the membrane with a higher Δ because probably the swelling-deswelling process has a major influence on the structure, already weakened by decomposition of the C–F bonds and the sulfonic acid groups.

In the diffusion region, ruled by mass transfer loss, the cell potential losses are similar and influenced by the greater resistive behavior of the polarization curves.

Anyhow, the untreated membrane shows the highest cell potential values at the BoT and the highest or the same values of N-HNO3 at EoT.

**Figure 10:**
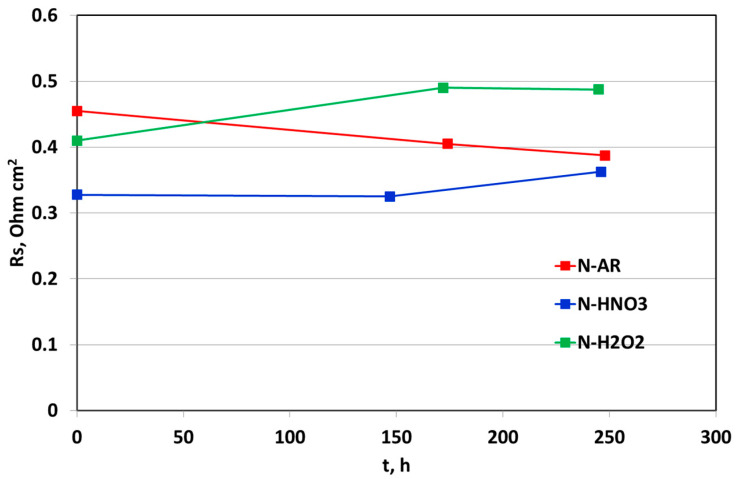
Trend of Rs measured from EIS as a function of time for N-AR (red squares), N-HNO3 (blue squares) and N-H2O2 (green squares) membranes.

Similarly, the ionic resistance R_s_, was recorded at BoT at around 145–174 h and at EoT. Rs, determined through EIS, is the intercept at high frequency of x-axis in a Nyquist plot and is mainly attributed to the membrane resistance. In Figure 10, the R_s_ values obtained at 0.7 V are reported. Again, N-H2O2 membrane shows a remarkable increase of resistance, confirming the membrane deterioration. N-HNO3, despite having a very low resistance at BoT, increases its resistance after about 145 h, until this value approaches to that of the untreated membrane at EoT. N-AR membrane gradually decreases its ionic resistance because it increases its wettability during fuel cell operation.

**Figure 11:**
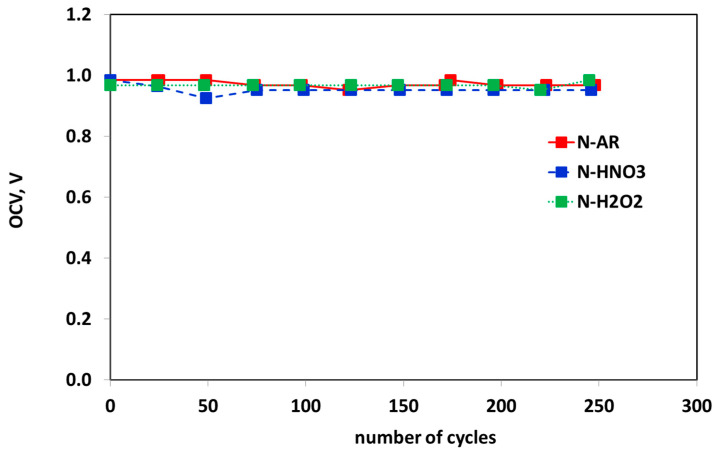
OCV trend during ASTs for N-AR (red squares), N-HNO3 (blue squares) and N-H2O2 (green squares) membranes.

In Figure 11, the OCV trend as a function of cycles number is shown. All the three membranes are sufficiently stable and the OCV remains constant in the whole AST.

**Figure 12:**
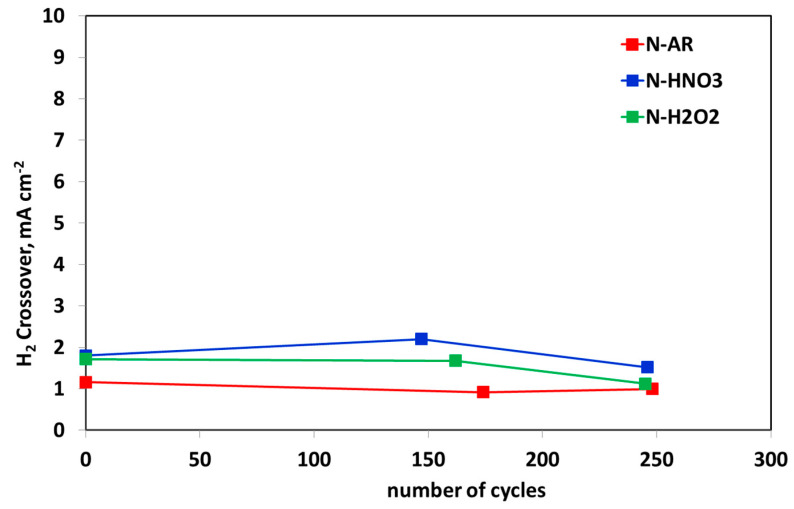
H_2_ crossover trend during ASTs for N-AR (red circles), N-HNO3 (blue squares) and N-H2O2 (green triangles) membranes.

In Figure 12, H_2_ crossover trend during ASTs is reported. This is an important parameter, responsible for the fuel cell failure. In fact, DoE fixes a value ≤2 mA·cm^−2^ as an acceptable limit for H_2_ crossover of the membranes in the fuel cell applications and ≤15 mA·cm^−2^ for the chemical and mechanical durability [31]. The H_2_ crossover of all membranes is enough low (≤2 mA·cm^−2^) for the entire duration of AST, except a peak of N-HNO3 membrane at 144 cycles. In any case, such membrane records the highest values up to EoT, while the untreated membrane N-AR is the membrane with the lowest H_2_ crossover.

Finally, the un-treated membrane (N-AR) results to be the best compromise between the chemical-physical and electrochemical properties, with a reduced swelling, a better mechanical resistance, good resistance to degradation in the fuel cell operations and, above all, with the lowest H_2_ crossover. This means that the laborious operations necessary for the chemical pre-treatments do not represent an added value for effectiveness of the membrane and its yield. Moreover, these treatments, differently performed in several labs, slightly change the membrane properties and are warily responsible for the scattering of the published data. Therefore, they could produce discrepant and not easily comparable results.

## 4. Conclusions

The commercial membrane Nafion™ NR212 has undergone two different pre-treatments before of the use. These treatments are usually applied to the membranes to oxide the impurities and/or to enhance the hydrophilicity and the IEC. The study carried out, in terms of chemical-physical and electrochemical properties, on the pre-treated and *as received* membranes, has shown that the pre-treated membranes have a higher hydrophilicity, but, on the other hand, they have a lower mechanical resistance, a higher swelling and dimensional variations that are detrimental for the fuel cell operations. These characteristics, in fact, affect the electrochemical performance and, especially, the cell degradation since H_2_ crossover increases through the membranes. The good results obtained using the untreated commercial NR212 membrane urges the use of commercial membranes without chemical pre-treatments, if not explicitly suggested by the producer, thus avoiding non reliable results, which are difficult to compare among them.

## Figures and Tables

**Table 1:** Main properties of Nafion™ NR212 membrane as reported by Ion Power^®^ [13].

Typical Thickness (µm)	50.8		
Basis Weight (g·m^−2^)	100		
**Physical Properties ^1^**	**MD ^2^**	**TD ^2^**	**Test Method**
Tensile strength, Max (MPa)	32	32	ASTM D882
Non-standard modulus, MPa	266	251	ASTM D882
Elongation to break, %	343	352	ASTM D882
**Other Properties**			
Specific gravity	1.97		Foonote ^1^
Available acid capacity, meq·g^−1^	0.92 min.		Foonote ^3^
Total acid capacity, meq·g^−1^	0.95–1.01		Foonote ^4^
Hydrogen crossover, mL·min^−1^·cm^−2^	< 0.010		Foonote ^5^
**Hydrolytic Properties**			
Water content, % water ^6^	5.0 ± 3.0		ASTM D570
Water uptake, % water ^7^	50.0 ± 5.0		ASTM D570
Linear expansion, % increase ^8^ from 50% Relative Humidity (RH), 23 °C (73 °F)			
to water soaked, 23 °C (73 °F)	10		ASTM D576
to water soaked, 100 °C (212 °F)	15		ASTM D576

^1^ Measurements taken with membrane conditioned to 23 °C (73 °F), 50%RH. ^2^ Where specified, MD—machine direction, TD—transverse direction. Condition state of membrane given. ^3^ A base titration procedure measures the equivalents of sulfonic acid in the polymer and used the measurements to calculate the available acid capacity of the membrane (acid form). ^4^ base titration procedure measures the equivalents of sulfonic acid in the polymer and used the measurements to calculate the total acid capacity or equivalent weight of the membrane (acid form). ^5^ Hydrogen Crossover measured at 22 °C (72 °F), 100%RH and 50-psi delta pressure. This is not a routine test. ^6^ Water content of membrane conditioned to 23 °C (73 °F) and 50%RH (dry weight basis). ^7^ Water uptake from dry membrane to conditioned in water at 100 °C (212 °F) for 1 h (dry weight basis). ^8^ Average of MD and TD. MD expansion is similar to TD expansion for NR membranes.

**Table 2:** IEC experimental values.

Membrane	IEC, meq/g
N-AR	1.03
N-HNO3	0.99
N-H2O2	1.0

**Table 3:** Cell voltage and percentage variation Δ obtained at Beginning of Test (BoT) and End of Test (EoT) at three different current densities.

Membrane	Cell Potential, V @ 0.1 A/cm^2^			Cell Potential, V @ 0.5 A/cm^2^			Cell Potential, V @ 1.0 A/cm^2^		
	BoT	EoT	Δ, %	BoT	EoT	Δ, %	BoT	EoT	Δ, %
N-AR	0.808	0.786	**−2.7**	0.703	0.670	**−4.7**	0.563	0.504	**−10.8**
N-HNO3	0.803	0.786	**−2.1**	0.670	0.654	**−2.4**	0.554	0.504	**−9.0**
N-H2O2	0.803	0.780	**−2.8**	0,687	0.620	**−9.7**	0.538	0.471	**−12.4**
